# ID 355 - Ampliação do Número de Fitoterápicos Cadastrados na Base Nacional de Dados de Ações e Serviços da Assistência Farmacêutica no SUS: impulsionando a visibilidade e a utilização de produtos à base de plantas medicinais no SUS

**DOI:** 10.5327/2237-9622.2025.v34s1.355

**Published:** 2025-11-25

**Authors:** Ellen Tanus Rangel, A. P. Barbosa, B. Morgado, R. C. de Almeida, V. C. Doneida, E. B. Farias, R. Poloni, B. B. Barreto, M. A. Pereira

**Keywords:** movimentação de fitoterápicos, Bnafar, PNPMF

## Abstract

**Introdução:**

A Política Nacional de Plantas Medicinais e Fitoterápicos (PNPMF) é essencial nas políticas públicas brasileiras, promovendo saúde, proteção ambiental e desenvolvimento sustentável. Seu objetivo é garantir o acesso seguro e o uso responsável de plantas medicinais, fortalecendo cadeias produtivas e estimulando a inovação. O Programa Nacional, alinhado à PNPMF, define ações para diversos órgãos, com significativa participação do Ministério da Saúde e da Secretaria de Ciência, Tecnologia, Inovação e do Complexo Econômico-Industrial da Saúde (Sectics). Cumpre informar que a Base Nacional de Dados de Ações e Serviços da Assistência Farmacêutica (Bnafar) é o principal instrumento para gerar indicadores que devem apoiar a tomada de decisões dos gestores das três esferas do governo. Segundo dados de movimentação de fitoterápicos extraídos da Bnafar, identificou-se que a maioria daqueles produzidos localmente, em especial nas Farmácias Vivas e Farmácias de Manipulação, não possui cadastro na base nacional, comprometendo a geração das informações essenciais para a gestão da Assistência Farmacêutica. Dito isso, é urgente a inserção de novos fitoterápicos na Bnafar. Objetivo: apresentar ação desenvolvida pela equipe gestora da PNPMF (alocada no Departamento de Assistência Farmacêutica e Insumos Estratégicos do Ministério da Saúde – DAF/MS), para a ampliação do número de fitoterápicos na Bnafar.

**Método:**

Levantamento descritivo das responsabilidades e ações institucionais de diferentes departamentos da Secretaria de Ciência, Tecnologia, Inovação e do Complexo Econômico-Industrial da Saúde.

**Resultados:**

Considerando que a inserção de fitoterápicos na Bnafar depende de cadastro prévio no Catálogo de Materias (Catmat), do Sistema Integrado de Administração de Serviços Gerais (Siasg), e que a inserção de itens de saúde no Catmat é realizada pela equipe de catalogadores do Ministério da Saúde, alocada no Departamento de Economia e Desenvolvimento em Saúde (Desid), a equipe gestora da PNPMF encaminhou ao referido Departamento uma lista contendo 99 fitoterápicos a serem cadastrados. Cumpre ressaltar que a lista foi elaborada em consonância com as solicitações já recebidas pela Coordenação-Geral de Assistência Farmacêutica Básica, de diferentes Secretarias de Saúde, além de estar alinhada à Relação Nacional de Plantas Medicinais de Interesse ao Sistema Único de Saúde (ReniSUS). Por fim, após a realização do cadastro no Catmat, a equipe técnica do DAF realizou a inserção dos fitoterápicos nos sistemas da Bnafar.

**Conclusão:**

A PNPMF orienta os serviços de saúde da União, dos estados e municípios, destacando a autonomia dos entes federativos para decisões no Sistema Único de Saúde (SUS). Cumpre destacar a importância de novos cadastros de fitoterápicos na Bnafar, além do envio de dados referentes à movimentação desses ao Ministério da Saúde, a fim de que possam subsidiar as ações dos gestores locais e federais no que se refere à Assistência Farmacêutica em Plantas Medicinais e Fitoterápicos. Além disso, a criação de um fluxo contínuo para o recebimento de informações sobre fitoterápicos produzidos localmente vem sendo elaborado e deverá ser implantado em breve.

